# Mitochondrial Dysfunctions in Neurodegenerative Diseases: Relevance to Alzheimer's Disease

**DOI:** 10.1155/2014/175062

**Published:** 2014-05-12

**Authors:** Jana Hroudová, Namrata Singh, Zdeněk Fišar

**Affiliations:** Department of Psychiatry, First Faculty of Medicine, Charles University in Prague and General University Hospital in Prague, Ke Karlovu 11, 120 00 Prague 2, Czech Republic

## Abstract

Mitochondrial dysfunctions are supposed to be responsible for many neurodegenerative diseases dominating in Alzheimer's disease (AD), Parkinson's disease (PD), and Huntington's disease (HD). A growing body of evidence suggests that defects in mitochondrial metabolism and particularly of electron transport chain may play a role in pathogenesis of AD. Structurally and functionally damaged mitochondria do not produce sufficient ATP and are more prominent in producing proapoptotic factors and reactive oxygen species (ROS), and this can be an early stage of several mitochondrial disorders, including neurodegenerative diseases. Mitochondrial dysfunctions may be caused by both mutations in mitochondrial or nuclear DNA that code mitochondrial components and by environmental causes. In the following review, common aspects of mitochondrial impairment concerned about neurodegenerative diseases are summarized including ROS production, impaired mitochondrial dynamics, and apoptosis. Also, damaged function of electron transport chain complexes and interactions between pathological proteins and mitochondria are described for AD particularly and marginally for PD and HD.

## 1. Introduction

Alzheimer's disease (AD) is the most common neurodegenerative disorder marked by progressive loss of memory and impairment of cognitive ability. AD can be classified into two forms: sporadic AD (SAD), where aging represents the main risk factor, in the vast majority of cases, and familial form of AD (FAD), where rare gene mutations have been identified [1, 2]. Both SAD and FAD patients share common clinical and neuropathological features including loss of neurons, intracellular neurofibrillary tangles (aggregates of hyperphosphorylated tau protein), and extracellular senile plaques, composed of *β*-amyloid (A*β*) deposits, which are derived from the proteolytic processing of the amyloid precursor protein (APP) [3]. According to a body of evidence, A*β* increases the neuron vulnerability to oxidative stress and impairments of electron transport chain (ETC) [4]. Pathologically, AD is featured by changes observed mostly in neocortex, hippocampus, and other subcortical regions essential for cognitive functions. Reduction in a variety of higher cortical functions—memory, orientation, and judgment—is evident [5].

## 2. Mitochondrial Involvement in Neurodegenerative Diseases

The series of events that lead to neurodegeneration are intricate. Various neurodegenerative disorders manifest with different symptoms and affect different parts of the brain. Mitochondrial dysfunctions are considered as conjunctive features, a point of convergence to different pathological pathways.

The mitochondria are cytoplasmic organelles in eukaryotic cells that are responsible for most of energy supply of cells. Besides, they are critical regulators of cell death and a key feature of neurodegeneration [6], and they play important role in cell processes, signaling pathways, calcium homeostasis, cell cycle regulation, apoptosis, reactive oxygen species (ROS) production, and thermogenesis [7]. The mitochondrial dysfunction, increased ROS production, and oxidative damage are responsible for numerous neurodegenerative disorders. Apoptosis and excitotoxicity are the two significant grounds of neuronal cell death and the role of mitochondria is crucial in both the cases [8]. Increased ROS production in neurodegenerative process might affect mitochondrial parameters and also ATP production, membrane potential, permeability transition pore (MPTP) activation, and calcium uptake. These changes can lead and result in neuronal damage. The first evidence of involvement of mitochondria in pathogenesis of neurodegenerative process was reported when complex I deficiency was detected in substantia nigra and platelet mitochondria of patients with Parkinson's disease (PD) [9, 10]. Further strong evidences were found for ETC deficiencies: complex I and cytochrome *c* oxidase (complex IV, COX) in AD and complexes II and III in Huntington's disease (HD) [11].

Biochemical analysis of postmortem AD brains found impaired function of the citric acid cycle enzymes, pyruvate dehydrogenase, *α*-ketoglutarate dehydrogenase, and isocitrate dehydrogenase. These changes correlated with the clinical state, and the function of enzymes could be related to diminished brain metabolism [12].

## 3. Impaired Mitochondrial Dynamics

Mitochondria are highly dynamic organelles, ranging from giant tubular networks to small round entities through rapid and reversible fission and fusion processes [13]. Fusion is mediated by large GTPase proteins such as optic atrophy factor 1 (OPA1) responsible for inner membrane fusion and mitofusin 1 (Mfn1) and mitofusin 2 (Mfn2) responsible for outer membrane fusion. Fusion is responsible for the proper distribution of mitochondrial components such as lipid membranes, oxidative phosphorylation complexes, and mitochondrial DNA (mtDNA). Fission plays an important role in the proper assembly of mitochondrial electron transport chain complexes; it is mediated by dynamin-related protein-1 (Drp1, GTPase), human fission protein 1 (Fis1), mitochondrial fission factor, and mitochondrial dynamics proteins (MiD49/MiD51) [14, 15]. Alteration in the expression of mitochondrial fusion-fission proteins can result in altered mitochondrial distribution [16].

Mitochondria failure might arise from a deficit dynamic balance of mitochondrial fission and fusion, and in AD it is greatly shifted towards fission and it could result in the dysfunctional mitochondria of damaged neurons. Immunoblot analysis found that expression of APP affected mitochondrial fusion/fission proteins; Drp1, OPA1, Mfn1, and Mfn2 were reduced, whereas Fis1 was significantly increased in AD [17, 18]. In mouse model of AD, mitochondrial dynamics was impaired; decreased mitochondrial anterograde movement, increased mitochondrial fission, decreased fusion, and defective mitochondrial functions were observed [19]. In human fibroblasts, from sporadic AD patients, mitochondrial distribution was characterized by elongated mitochondria accumulated in perinuclear areas [20]. Further this study demonstrated that elevated oxidative stress and increased A*β* production are potential factors causing Drp1 reduction [20].

Tau mutation P301L cells (SY5Y cells overexpressing P301L tau protein) demonstrated complex I deficit and decreased ATP levels [21]. Phosphorylated tau (pTau) and A*β* cause enhanced nitrosylation of Drp1 protein, which leads to increased mitochondrial fission and neurodegeneration [22]. Cells deficient in mitochondrial fusion showed loss of mitochondrial membrane potential (Δ*ψ*_*m*_) and reduced mitochondrial respiration [23]. Interestingly, reduced OPA1 was shown to induce spontaneous cytochrome *c* (cyt *c*) release and to accelerate cyt *c* release by apoptotic stimuli [24]. In summary, the following were reported: increased mitochondrial fission and decreased fusion, increased A*β* and pTau interaction with the mitochondrial fission protein Drp1, likely leading to increased mitochondrial fragmentation, impaired axonal transport of mitochondria, and synaptic degeneration in neurons affected by AD [25, 26].

AD, PD, and HD are associated with the accumulation of amyloid fibrils [27, 28]. Soluble oligomers of amyloid proteins are able to permeabilize cellular membranes and lipid bilayers and disrupt membrane functions; the mechanism of disruption is not clearly understood. They can be inserted into membranes, affect dielectric membrane properties and disrupt normal ion gradients, and/or inactivate normally functioning proteins [28, 29]. Amyloid oligomers increased conductance in a conformation-specific shape; it is dependent on the concentration of oligomers and can be reversed by antioligomer antibody.

In HD, mutant huntingtin interacts with Drp1 and related GTPases and causes excessive mitochondrial fragmentation and abnormal distribution of mitochondria. Altered mitochondrial morphogenesis, increased mitochondrial fission, and reduced fusion together with mitochondrial loss are linked to neuronal dysfunctions and cell death [30–32]. Abnormal dynamics of mitochondria results in the loss of ETC complex function.

In PD, parkin interacts with alpha-synuclein and contributes to pathophysiology [33, 34]. Hereditary form of PD is related to genes for PINK1 and parkin, which are important for mitochondrial integrity. These proteins have been suggested to promote mitochondrial fission and to inhibit fusion [35, 36]. PINK and parkin probably regulate mitochondrial dynamics and promote the turnover of damaged mitochondria [37].

## 4. Mitochondrial ROS and Apoptosis

The imbalance between cellular production of ROS and the ability of cells to efficiently defend against them is called “oxidative stress.” Oxidative stress is linked to neurodegenerative diseases and aging processes; it can be the source of cellular damage causing necrotic or apoptotic cell death since the ROS oxidize vital cellular components, lipids, proteins, and nucleic acids [38].

Impaired function of oxidative phosphorylation (OXPHOS) may cause disturbances of energy metabolism, which are frequently observed in AD. Impaired energy metabolism results in decreased respiratory control ratio as well as ATP levels [39]. There are many possible mechanisms for reduced oxidation rates and ATP production rates that do not include a defect of respiratory chain enzymes [40].

ROS have their role in intracellular signalling and regulation of signal transduction [2]. ROS seem to be the key factors in brain aging processes and disturbed mitochondrial respiration, accompanied by increased ROS production, significantly contributes to functional changes in brain during aging. Complex I and complex III are considered to be the primary source of ROS in brain under physiological conditions, as well as in pathological processes (e.g., neurodegenerative disorders). Complex I releases superoxide (O_2_^•−^) to matrix, and complex III can release O_2_^•−^ to both sides of the inner mitochondrial membrane. By superoxide dismutase, O_2_^•−^ can be converted to hydrogen peroxide (H_2_O_2_), which permeates by membranes and can be source of highly reactive hydroxyl radical. Physiologically generated H_2_O_2_ and O_2_^•−^ from ETC are dependent on the magnitude of proton-motive force (Δ*p*) and the respiratory state of mitochondria [41]. State 4 is characterized with high rate of ROS; on the contrary, states 3 and 5 produce minimum of ROS [42].

Both disturbed production and detoxification of ROS participate in pathophysiological effects of mitochondrial dysfunctions [43, 44]. Defective mitochondria release large amounts of ROS; similarly, decline of antioxidative enzyme activities (e.g., in the elderly) enhances ROS formation [45]. Negative results of ROS can affect respiratory chain; complexes I, III and COX seem to be the most affected, whereas function of complex II appears to be unchanged [46, 47].

Mitochondria play a pivotal role in intrinsic pathway of apoptosis [48]. During apoptosis, mitochondrial network is disintegrated and the outer mitochondrial membrane is permeabilized, which leads to release of several apoptotic proteins, cyt *c* included. There are interrelated mitochondrial pathways that facilitate cell death: (i) opening of MPTPs can lead to mitochondrial swelling and cell death through apoptosis or necrosis; (ii) increase in the permeability of the mitochondrial membrane causes leak of apoptotic factors (second mitochondria-derived activator of caspases (Smac) and cyt *c*), which trigger the caspase cascade leading to apoptosis; and (iii) release of caspase-independent death effector, apoptosis-inducing factor (AIF), triggers chromatin condensation and DNA degradation [49]. Mitochondria undergo fragmentation during apoptosis before caspases are activated [50]. In apoptotic cells rapid loss of the inner Δ*ψ*_*m*_ is accompanied by ROS production.

Recently, attention is paid to the ROS-induced damage of ETC complexes mediated by a peroxidation and oxidative damage of cardiolipin [22, 51, 52]. Membrane lipids, cardiolipin mainly, are both required for the stability of respiratory supercomplexes and serve as a diffusion microdomain for the ubiquinone [53]. Cardiolipin plays also an active role in mitochondrial mediated apoptosis, can be oxidized, and interacts with cyt *c* and Bcl-2 proteins [54].

**Figure 1:**
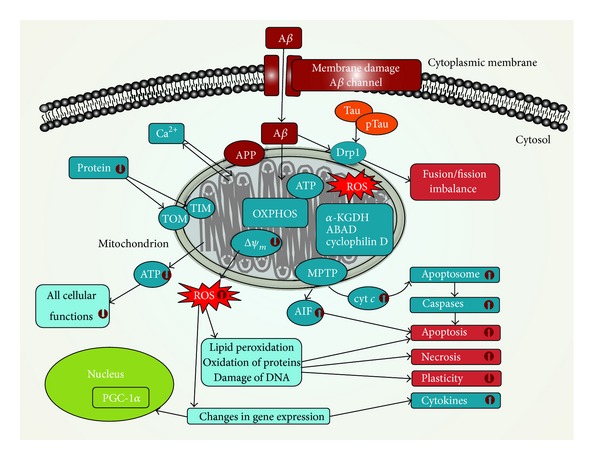
Mitochondrial dysfunctions in Alzheimer's disease. Amyloid-beta (A*β*) impairs the integrity of cytoplasmic membrane and causes mitochondrial dysfunctions. A*β* inhibits the activity of oxidative phosphorylation (OXPHOS) system, which can result in decrease of ATP production and increased reactive oxygen species (ROS) formation. Decreased ATP production leads to impairment of ATP-dependent processes, where all cellular functions are involved. Decrease of mitochondrial membrane potential (Δ*ψ*_*m*_) is followed by opening of mitochondrial permeability transition pores (MPTPs). Release of cytochrome *c* (cyt *c*) and other proapoptotic factors from the intermembrane space of mitochondria induces the formation of apoptosome and consequently triggers activation of caspases and apoptosis. Apoptosis inducing factor (AIF) is a proapoptotic factor released by mitochondria. Disengaged AIF is transported into nucleus and triggers caspases-independent apoptosis. Phosphorylated tau protein (pTau) and A*β* cause enhanced nitrosylation of dynamin-related protein-1 (Drp1) leading to impaired mitochondrial dynamics, increased mitochondrial fission, and neurodegeneration. Further, A*β* inhibits the import of proteins into mitochondria and reduces activity of mitochondrial amyloid-beta binding alcohol dehydrogenase (ABAD), *α*-ketoglutarate dehydrogenase complex (*α*-KGDH), and cyclophilin D. Ability of mitochondria to handle Ca^2+^ is impaired by A*β* and A*β* precursor protein (APP); consequently overload of mitochondrial calcium leads to decrease of Δ*ψ*_*m*_, opening of MPTPs, releasing of proapoptotic factors, increased ROS production, and decreased ATP production. PGC-1—peroxisome proliferator-activated receptor-gamma coactivator-1-alpha; TIM—translocase of the inner membrane; TOM—translocase of the outer membrane.

In AD, membrane-associated oxidative stress, increased free radical production, and perturbed Ca^2+^ homeostasis have been observed. Increased mitochondrial permeability and cyt *c* release, which is promoted by A*β* and alpha-synuclein oligomerization and polymerization, trigger the opening of MPTP leading to apoptosis [55]. In addition, COX activity is reduced and neurons exhibit mitochondrial damage and apoptosis. However, the cause of mitochondrial alterations in Alzheimer's disease remains unknown. Processes of mitochondrial impairment in AD are shown in Figure 1.

## 5. Mitochondrial DNA in AD

Changes of mtDNA are particularly responsible for aging of phenotypes. Defects in mtDNA have been found also in non-AD elderly persons; many tissues have lower respiratory function and decreased COX activity [56]. Brain mtDNA in AD has more oxidative damage beyond that due to aging, which can lead to increased mutations/deletions and postgenomic problems with transcriptional regulation [57]. Changes of the expression of mitochondrial and nuclear genes, encoding parts of COX and complex I enzymes, contribute to alterations of oxidative metabolism in AD [58]. Downregulation of mitochondrial genes in complex I was found in early as well as in definite AD brain specimens [59]. Studies reported decreased complex I activity in AD brains [60, 61], and gene expression of ND4 subunit of complex I was found decreased in temporal cortex of AD patients [62]. Differential expression of mitochondrial genes encoding complex I, COX, and complex V was determined in AD brains [59]. Likely, mtDNA does not play a primary role in the AD pathogenesis but can be involved subsequently [63]. Increased gene expression of COX might be a result of increased oxidative damage and early alteration of mitochondrial function in surviving neurons. Expressions of mitochondrial encoded COX I subunit and nuclear encoded COX IV were examined in hippocampi of AD patients. Level of mitochondrial encoded COX IV correlated with the amount of hyperphosphorylated tau protein accumulated in certain hippocampal area but not with the amount of accumulated A*β* [64]. Another study found the distribution of amyloid plaques distinct from COX deficient neurons in hippocampus [65]. In addition to these results, COX-deficient mice exhibited significantly fewer amyloid plaques accompanied by a reduction of *β*-secretase, A*β*-42, and oxidative damage [66].

Expression of mitochondrial and nuclear genes, encoding parts of COX and complex I, was examined in selected brain areas from AD patients and controls. Altered proportions between subunits of COX, COX II, and COX IV mRNAs were observed in the AD brains. Changes of proportions between these subunits may contribute to kinetic perturbation documented for COX in AD. Decrease of ND4 and ND15 mRNAs (encoding subunits of complex I) was observed in AD hippocampus and inferior parietal lobule, but not in cerebellum. These changes of genes encoding parts of complex I and COX may contribute to alterations of oxidative metabolism in AD [58].

Fusion-fission imbalance is related to altered mtDNA; mitochondrial fusion enables the exchange of mitochondrial content including mtDNA. Inhibition of fusion by Mfn2 knockout resulted in majority of mtDNA-lacking mitochondria [67].

## 6. Impairment of ETC in AD

Activity of COX was found to be reduced in platelets of AD patients [68]. Similarly, significantly decreased COX activity was observed in cortex of AD patients [69]. Another study confirmed the decreased activity in hippocampus of AD patients that suggests the anatomical specificity [70]. Mitochondrial deficiencies were found in platelets of AD patients indicating significant decline of complex III and COX activity [71]. It has been shown that acetylcholinesterase (AChE) was reduced; further, it was demonstrated that AChE could increase the A*β* activity [72, 73].

ETC activities of human lymphocytes were evaluated in AD patients, and increased complexes II and IV activities were observed; this might be a compensatory mechanism to supply the energy [74]. Evidences of ETC dysfunctions in AD are summarized in Table 1.

Distinct mitochondrial abnormalities associated with neurodegenerative diseases culminate in oxidative stress, energy dysfunction, and aberrant homeostasis of cytosolic calcium [75]. System of OXPHOS does not respond to thermodynamic equilibrium but embodies a rate of uncoupling. Lower Δ*ψ*_*m*_ can result in hydrolysis of cytoplasmic ATP; high Δ*ψ*_*m*_ leads to proton leak and increased uncoupling. ROS overproduction, decreased Δ*ψ*_*m*_, and Ca^2+^ dependent increase of MPT lead to apoptosis [42]. Decreased rates of electron transfer were identified as mechanism of mitochondrial dysfunction on aging, and complex I and COX were found decreased upon aging [76]. Inhibition of complex III and COX is required to increase glutamate release Ca^2+^ independent [77]. Partial inhibition of complex I activity reduced nerve terminal oxygen consumption and increased glutamate release from depolarized synaptosomes [78].

## 7. Conclusions

Mitochondrial dysfunctions involved in pathophysiology of neuropsychiatric disorders include disturbances in OXPHOS, increased mitochondrial DNA (mtDNA) deletions, mutations or polymorphisms, impaired calcium signalling, and impaired energy metabolism as well as interactions with disease specific proteins (e.g., A*β*, parkin, PINK1, alpha-synuclein, and huntingtin). Mitochondrial pathology could be an important factor in the manifestation of clinical symptoms of neurodegenerative disorders; thus therapeutic approaches to strengthen mitochondrial functions could be certainly meaningful.

Evidence supports using antioxidants and other mitochondria-targeting compounds with potential efficacy in AD, for example, carnitine, vitamin C, vitamin E, alpha-lipoic acid, coenzyme Q_10_, methylene blue, piracetam, simvastatin,* Ginkgo biloba*, curcumin, and omega-3 polyunsaturated fatty acids [79, 80]. Targeting mitochondrial proteins might represent a novel therapeutic strategy against AD; for example, several mitochondria targeted antioxidants have been developed. Shift in mitochondrial dynamics (extensive fission) in AD negatively impacts all aspect of mitochondrial function and may be critical to AD pathogenesis. Therefore, strategies to modify abnormal mitochondrial dynamics may be an attractive therapeutic intervention target for AD. Therapeutics that target to reduce the expression of the mitochondrial fission protein Drp1, A*β*, and pTau may protect neurons from toxic insults of these factors and their interactions.

## Figures and Tables

**Table 1:** Evidences of ETC dysfunctions in AD.

Biological model	Affected mitochondrial function	Reference
Lymphocyte mitochondria of AD patients	Higher oxidative (oxidation of pyruvate-malate, glycerol-3-phosphate) and enzymatic activities (I, II, and III) were found in AD patients treated with rivastigmine rather than untreated AD patients.	[81]

Transgenic mice crude forebrain	Tau-dependent deregulation of complex I and A**β**-dependent deregulation of complex II, synergistic effects of deregulation in AD mice, and reduction in mitochondrial membrane potential.	[82]

Lymphocytes	Alterations in respiratory chains—activity of complexes II and IV was higher.	[74]

Platelets and postmortem motor cortex and hippocampus from AD patients	COX but not F_0_F_1_-ATPase is a mitochondrial target in AD, in both a brain association area and platelets. A reduced COX activity may make the tissue vulnerable to excitotoxicity or reduced oxygen availability.	[83]

Posterior cingulate (area 23) cortex	The findings suggest a decrement of cytochrome oxidase in posterior cingulate cortex, with progressive reduction within the superficial laminas linked to disease duration.	[84]

Platelet and lymphocyte mitochondria	Significant declines in complexes III and IV.	[71]

Postmortem brain tissue	Complex I and complexes II-III slightly decreased in occipital cortex, and COX decreased significantly in cortical areas (frontal, temporal, parietal, and occipital).	[69]

Autopsied human brain mitochondria	AD brain mitochondria demonstrated a generalized depression of activity of all electron transport chain complexes. This depression was most marked in COX activity (*P* < 0.001). Concentrations of cytochromes b, c1, and aa3 were similar in AD and controls. The electron transport chain is defective in AD brain, and the defect centers around COX.	[61]

Subcortical centers: thalamus, the globus pallidus, the red nucleus, and the locus coeruleus	Changes of the mitochondrial cristae, accumulation of osmiophilic material and decrease of their size, and mitochondrial alterations were particularly prominent in neurons, which showed loss of dendritic spines and abbreviation of the dendritic arborization.	[85]

Human seven brain regions (cerebellum, frontal, temporal, occipital, parietal cortices, thalamus, and caudate nucleus)	Complex III core protein was significantly reduced in the temporal cortex of AD patients.	[86]

Autopsied brain mitochondria	COX activity reduced in frontal, temporal, and parietal cortices and normal COX activity reduced in occipital cortex.	[87]

Human seven brain regions (cerebellum, frontal, temporal, occipital, parietal cortices, thalamus, and caudate nucleus)	Complex I 24-kDa subunit was significantly reduced in temporal and occipital cortices. Complex I 75-kDa subunit was significantly reduced in parietal cortex region of brain.	[88]

Human brain: frontal cortex, temporal cortex, hippocampus, and cerebellum	Specific defect of COX in the confined brain regions, suggesting anatomic specificity.	[70]

Human cytoplasmic hybrid (cybrid) neurons with incorporated platelet mitochondria	Significant changes in morphology and function; such changes associate with altered expression and distribution of dynamin-like protein (Dlp1) and mitofusin 2 (Mfn2), mitochondrial fission-fusion imbalances.	[89]

*In situ* nerve terminal and synaptosomal mitochondria of rats	High level of inhibition is required for glutamate efflux from nerve terminal.	[77]

Rat forebrain mitochondria	Loss of cyt *c* by mitochondria oxidizing NAD^+^-linked substrates results in a dramatic increase of ROS production and respiratory inhibition.	[90]

Mitochondria from brains of transgenic mice	A*β* progressively accumulates in mitochondria and is associated with diminished enzymatic activity of complex III and COX, reduction in the rate of oxygen consumption.	[91]

Human neuroblastoma cells (SH-SY5Y)	Increased complex III activity and decreased COX activity were found. Decreased respiratory control ratio and ATP levels.	[39]

Human blood platelets	ATP levels were reduced, while ROS were increased in AD patients. Platelet membrane fluidity, vitamin E, and cholesterol content were similar between effected and noneffected groups.	[92]

## Abbreviations

ABAD: Amyloid-beta binding alcohol dehydrogenase
AChE: Acetylcholinesterase
*α*-KGDH: *α*-ketoglutarate dehydrogenase
A*β*: *β*-amyloid
AD: Alzheimer's disease
AIF: Apoptosis-inducing factor
*β*-APP: *β*-amyloid precursor protein
COX: Cytochrome *c* oxidase, complex IV
cyt *c*: Cytochrome *c*
Drp1: Dynamin-related protein 1
ETC: Electron transport chain
FAD: Familial Alzheimer's disease
FA: Fatty acid
HD: Huntington's disease
H_2_O_2_: Hydrogen peroxide
MPT: Mitochondrial permeability transition
MPTP: Mitochondrial permeability transition pore
mtDNA: Mitochondrial DNA
O_2_^•−^: Superoxide radical
OPA1: Optic atrophy factor 1
OXPHOS: Oxidative phosphorylation
Δ*p*: Proton-motive force
PD: Parkinson's disease
ROS: Reactive oxygen species
SAD: Sporadic Alzheimer's disease
Smac: Second mitochondrial-derived activator of caspase
Δ*ψ*_*m*_: Mitochondrial membrane potential
PGC-1: Peroxisome proliferator-activated receptor-gamma coactivator-1-alpha
ROS: Reactive oxygen species
pTau: Phosphorylated tau protein
TIM: Translocase of the inner membrane
TOM: Translocase of the outer membrane.
